# Simultaneous silencing of two different *Arabidopsis* genes with a novel virus-induced gene silencing vector

**DOI:** 10.1186/s13007-020-00701-6

**Published:** 2021-01-06

**Authors:** Kunxin Wu, Yadan Wu, Chunwei Zhang, Yan Fu, Zhixin Liu, Xiuchun Zhang

**Affiliations:** Institute of Tropical Bioscience and Biotechnology, Chinese Academy of Tropical Agricultural Sciences/Key Laboratory of Biology and Genetic Resources of Tropical Crops, Ministry of Agriculture, Haikou, 571101 China

**Keywords:** *Turnip crinkle virus*, Virus-induced gene silencing, Visualizable indicator, AGO2, DCL4-mediated antiviral defense, DRB4-independent

## Abstract

**Background:**

Virus-induced gene silencing (VIGS) is a useful tool for functional characterizations of plant genes. However, the penetrance of VIGS varies depending on the genes to be silenced, and has to be evaluated by examining the transcript levels of target genes.

**Results:**

In this report, we report the development of a novel VIGS vector that permits a preliminary assessment of the silencing penetrance. This new vector is based on an attenuated variant of *Turnip crinkle virus* (TCV) known as *CPB* that can be readily used in *Arabidopsis thaliana* to interrogate genes of this model plant. A *CPB* derivative, designated *CPB1B*, was produced by inserting a 46 nucleotide section of the *Arabidopsis PHYTOENE DESATURASE* (*PDS*) gene into *CPB*, in antisense orientation. *CPB1B* induced robust *PDS* silencing, causing easily visible photobleaching in systemically infected *Arabidopsis* leaves. More importantly, *CPB1B* can accommodate additional inserts, derived from other *Arabidopsis* genes, causing the silencing of two or more genes simultaneously. With photobleaching as a visual marker, we adopted the *CPB1B* vector to validate the involvement of *DICER-LIKE 4 (DCL4)* in antiviral defense against TCV. We further revealed the involvement of *ARGONAUTE 2 (AGO2)* in *PDS* silencing and antiviral defense against TCV in *dcl2drb4* double mutant plants. These results demonstrated that *DOUBLE-STRANDED RNA-BINDING PROTEIN 4 (DRB4)*, whose protein product (DRB4) commonly partners with DCL4 in the antiviral silencing pathway, was dispensable for *PDS* silencing induced by *CPB1B* derivative in *dcl2drb4* double mutant plants.

**Conclusions:**

The *CPB1B*-based vector developed in this work is a valuable tool with visualizable indicator of the silencing penetrance for interrogating *Arabidopsis* genes, especially those involved in the RNA silencing pathways.

## Background

RNA silencing is a generic term covering a number of mechanistically related phenomena occurring in eukaryotic organisms ranging from fungi to humans. Briefly, silencing is triggered by double-stranded RNA (dsRNA) or partially double-stranded hairpin RNA, which is digested by a dsRNA-specific RNase (Dicer or Dicer-like in plants) into small interfering (siRNAs) or micro (miRNAs) RNAs with length of 21–24 nucleotide (nt) [1, 2]. In addition to Dicer-likes (DCLs), this dsRNA processing step frequently requires one member of the dsRNA-binding protein (DRB) family [3]. Once produced, siRNAs and miRNAs are recruited by Argonaute proteins (AGOs) into RNA-induced silencing complexes for the direct degradation or the translational repression of other homologous RNAs or the modification of homologous chromatin DNA [1, 2].

The virus-induced gene silencing (VIGS) system takes advantage of this defense system to silence endogenous RNA sequences that are homologous to a sequence engineered into the viral genome, which generates the dsRNA that mediates silencing. As a tool, VIGS has many advantages over conventional techniques, such as independent genetic transformation, easy manipulation, high effectiveness, and suitability for the large-scale functional analysis of genes and for analyzing genes that cause lethal phenotype. In addition, the effect of silencing can be monitored within a short time after inoculating plants with the virus. Given these features, VIGS is an attractive reverse genetic tool for functional genomics in plants [4–8]. In the past decade, a number of viral genomes has been modified as a powerful reverse genetic tool for the functional characterization of genes in plants, such as *Tobacco rattle virus* [9], *Apple latent spherical virus* [10, 11], *African cassava mosaic virus* [12], and *Cucumber mosaic virus* [13]. However, most of the reported VIGS vectors only silence a single gene, and the VIGS vectors with visualizable indicator to evaluate the early penetrance of genes for reverse genetics are lacking.

This work aims to develop an efficient and stable viral vector with visualizable indicator that permits a preliminary assessment of the silencing penetrance, and use as a tool to study genes that may play a role in gene silencing pathways. The attenuated variant of *Turnip crinkle virus* (TCV) known as *CPB* which has compromised ability of TCV capsid protein (CP) to suppress RNA silencing is chosen for testing and further modifications [14]. Our previous work has shown that the *CPB-CC-PDS* generated by fusing 90 nt *Arabidopsis PDS* gene to *CPB* can induce modest *PDS* silencing in wild type *Arabidopsis* plants and several *Arabidopsis* mutant plants such as *dcl2*, *dcl4*, *drb4*, *dcl2drb4*, *dcl4drb4* [15, 16], thereby providing a visual indicator for the silencing inducing capability of this viral mutant. Compared with other VIGS vectors, *CPB* has a number of advantages. First, the TCV genome consists of just one positive (+) sense RNA, which is merely 4054 nt long and requires neither a 5′ cap nor a 3′ poly A tail, making the in vitro synthesis of infectious RNA extremely simple and cost-effective [17]. Second, the TCV replicates in the model plant *Arabidopsis* to produce extremely high levels of viral RNA, dsRNA, and viral siRNAs (vsiRNAs), simplifying the detection and purification processes [14–18].

In the current study, a *CPB* derivative, designated *CPB1B*, was produced by inserting a 46 nt section of the *Arabidopsis PDS* gene into *CPB*, in antisense orientation. We have demonstrated that *CPB1B* induced robust *PDS* silencing, causing easily visible photobleaching in systemically infected *Arabidopsis* leaves. More importantly, *CPB1B* can accommodate additional inserts, derived from other *Arabidopsis* genes, causing the silencing of two or more genes simultaneously. The optimal insertion size of the *CPB1B* is around 100 nt. With photobleaching as a visualizable indicator of the penetrance of VIGS, we adopted the *CPB1B* vector to validate the involvement of *DICER-LIKE 4 (DCL4)* in antiviral defense against TCV. Furthermore, we further revealed the involvement of *ARGONAUTE 2 (AGO2)* in *PDS* silencing and antiviral defense against TCV in *dcl2drb4* double mutant plants. Notably, *DOUBLE-STRANDED RNA-BINDING PROTEIN 4 (DRB4)*, whose protein product (DRB4) commonly partners with DCL4 in the antiviral silencing pathway, the results demonstrated DRB4 was dispensable for *PDS* silencing induced by *CPB1B* derivatives in *dcl2drb4* double mutant plants. These results indicated the *CPB1B*-based VIGS system as a valuable tool for interrogating *Arabidopsis* genes, especially those involved in the RNA silencing pathways. At the same time, this work opens a potential avenue for the development of VIGS vector through the synthesis of short fragments, which include several predicted siRNA sequences to silence two or more functional genes at the same time.

## Methods and materials

### Plant materials

The sources of knockout mutant *dcl4*, *dcl2*, and *dcl2drb4* plants were kind gifts from Feng Qu and have been described previously [16, 18]. All mutants were verified through genotyping. Uninfected *Arabidopsis* plants were reared in a growth room with 14 h of daylight and temperature of 22 °C. The *Arabidopsis* plants were inoculated with in vitro transcribed viral RNA when they were 3 to 4 weeks old. After inoculation the infected plants were moved into versatile environmental test chamber (SANYO) that is set at 18 °C, 14-h daylight with a light intensity of 160–190 μmol/m^2^/s.

### Generation of VIGS constructs

**Fig. 1:**
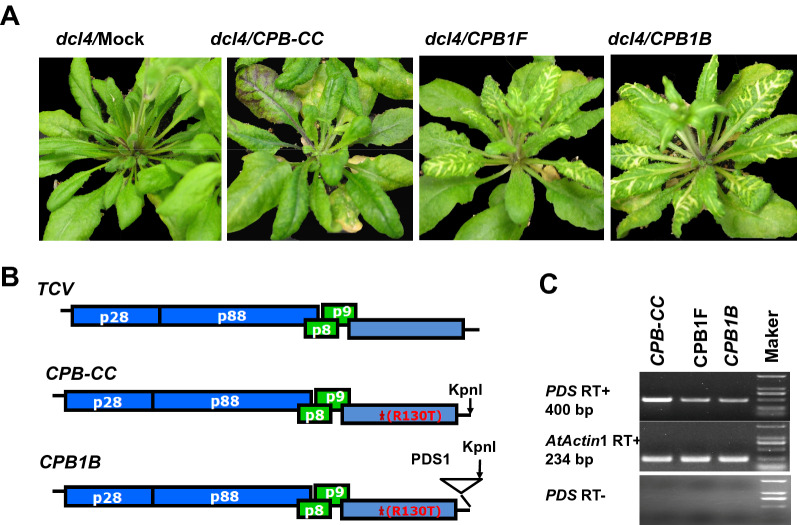
*CPB-CC*-based vectors harboring 46 nt *PDS* fragment inserted in both orientations can effectively trigger *PDS* silencing in *Arabidopsis*. **a** *PDS* silencing induced by *CPB-CC*-based vectors in *dcl*4 plants. Images are recorded at 25 dpi. At 11 dpi, the upper uninoculated leaves of CPB1F- or *CPB1B*-infected plants exhibit a photobleaching phenotype in the upper leaves, resulting from the reduction in the expression level of *PDS*. CPB1F and *CPB1B* represent a 46 nt *PDS* fragment inserted in the sense and the antisense orientations, respectively. **b** Diagrams of TCV, *CPB-CC*, and *CPB1B* constructs. The *CPB-CC* construct is produced by changing the AT dinucleotides to CC at nt 3807 to 3808, within the 3′ UTR of *CPB*, which has R130T mutation denoted by a red star, resulting in a new KpnI site. The *CPB1B* construct is produced by fusing 46 nt *PDS* fragment in *CPB-CC* in the antisense orientation. **c** Downregulation of *PDS* mRNA levels by CPB1F and *CPB1B* in *dcl*4 plants as determined using semiquantitative RT-PCR. The samples are collected at 14 dpi. The *AtActin*1 mRNA is used as a control to ensure that similar amounts of RNA are used in all reactions

The original TCV VIGS vector plasmid, *CPB*, was constructed in Dr. Qu’s lab at Ohio state University, as described in a previous study [14]. Briefly, *CPB* contained an arginine (R) to threonine (T) mutation at position no. 130 of the TCV CP that substantially compromised the ability of TCV CP to suppress RNA silencing [14]. *CPB* was further modified by changing the AT dinucleotide at nt 3807 to 3808 of the TCV genome to CC, creating *CPB-CC* with a new KpnI site immediately downstream of the TCV CP coding region (Fig. 1b) [15].

To determine the optimal orientation of the foreign insert in *CPB-CC*-based VIGS vector, the *PDS* fragment inserted in *CPB-CC-PDS* [15] was subjected to the software https://www.genscript.com/tools/sirna-target-finder to predict the siRNAs. In accordance with the results, one set of short complementary oligos containing the predicted siRNA sequence were synthesized. The complementary oligos in 1× T4 DNA ligase buffer were annealed in the Thermocycler with the following cycling conditions: 95 ℃, 5 min; 65 ℃, 5 min; 35 ℃, 5 min; and 25 ℃, 5 min. The resulting annealed dsDNA oligo was ligated into the *CPB-CC* digested and dephosphorylated using KpnI. The ligated plasmid was transformed into *Escherichia coli* (DH5α strain)-competent cells. The primers TCV-3334F and TCV-4000R (Additional file 1: Table S3) were used for colony screening. The orientation of the insertion was determined using the restriction enzymes EheI and KpnI. The resulting two *CPB-CC*-derived constructs were named *CPB1F* and *CPB1B*, which were inserted with *PDS* fragment in the sense (F) or antisense (B) orientations, respectively.

The primers CPB1B-F, CPB1B-102R, CPB1B-139R, CPB1B-215R (Additional file 1: Table S2) were designed to amplify 102-, 139-, and 215 nt *PDS* fragments to generate *CPB1B*-based VIGS vectors with different sizes of insert (Additional file 1: Fig. S2). The resulting fragments were ligated into KpnI-treated *CPB1B* through the Gibson assembly master mix (New England BioLabs) in accordance with the manufacturer’s description. The primers TCV-3334F and TCV-4000R (Additional file 1: Table S3) were used for colony screening.

For the construction of *CPB1B* vectors carrying the insert of *Arabidopsis DCL*4 and *AGO*2 gene respectively, the sequences of *DCL*4 (NM_122039.5), and *AGO*2 (NM_102866.3) were retrieved from NCBI. The reverse complement sequence of each gene was subjected to the software https://www.genscript.com/tools/sirna-target-finder to predict the siRNAs. The fragments with size of 100 ± 2 nt, which included at least one of the top five predicted siRNA sequences, was selected (Additional file 1: Table S1). The selected fragments were amplified from the *Arabidopsis* cDNA by using the primer sets AtDCL4A-F/AtDCL4A-R, AtDCL4B-F/AtDCL4B-R, AtAGO2A-F/AtAGO2A-R, and AtAGO2B-F/AtAGO2B-R (Additional file 1: Table S2). The resulting PCR products were cloned into KpnI-cut *CPB1B* through the Gibson assembly master mix mentioned above. Positive colonies were screened using primers TCV-3334F and TCV-4000R (Additional file 1: Table S3). Similarly, one 100 nt GUS fragment was selected (Additional file 1: Table S1) and amplified from the pCAMBIA1302 with primer set GUS-F/GUS-R (Additional file 1: Table S2) to generate a nontarget VIGS control. All constructs were sequenced to verify their identity.

### Infection of *Arabidopsis* with in vitro transcripts

The in vitro transcripts of TCV variants were produced using the TranscriptAid T7 HighYield Transcription Kit (Fermentas, Glen Burnie, MD) in accordance with the manufacturer’s instruction. The integrity and the concentration of the transcripts were examined using agarose gel electrophoresis. The inoculum was prepared by diluting the transcripts to 10 ng/μL by using the inoculation buffer (pH 9.2) containing 50 mM glycine, 30 mM K_2_HPO_4_, 1% bentonite, and 1% celite. 10 μL inoculum was used for mechanical inoculation with a gloved finger on each *Arabidopsis* leaf. Usually for each mutant and the wild type controls, at least six plants were infected with the infectious transcripts of clones described above, on three fully expanded leaves per plant.

### Reverse transcription-PCR (RT-PCR)

Total RNAs were isolated from the upper uninoculated *Arabidopsis* leaves which were about one centimeter long at 14 days postinoculation (dpi) using the TRIzol (Tiangen Biotech Beijing Co., Ltd) following the manufacturers protocol. Leaves from six different plants (one leaf per plant) were pooled before RNA extraction to minimize sampling errors. The first-strand cDNA was generated using reverse-transcription reactions through the FastQuant RT kit (KR106-01, Tiangen Biotech Beijing Co., Ltd) in accordance with the manufacturer’s instruction. PCR was carried out with the primer sets TCV-3334F and TCV-4000R to detect the genetic stability of the foreign inserts of *CPB1B*-based vectors in *Arabidopsis* (Additional file 1: Table S3).

### Semiquantitative RT-PCR

The semiquantitative RT-PCR was used to analyze the mRNA expression levels of the endogenous *PDS* genes in either *dcl2* or *dcl2drb4* plants inoculated with *CPB-CC* or *CPB1B*-based vectors at 14 dpi. Total RNAs and the first-strand cDNAs were obtained as described above. The expression level of the *PDS* gene was determined using primer sets AtPDS-1200F/AtPDS-1600R (Additional file 1: Table S3). The expression of the *AtActin1* gene determined using the primer set AtActin1-F/AtActin1-R (Additional file 1: Table S3) was referred as an internal control to normalize cDNA concentrations. PCR amplifications were performed for 28 cycles. Each PCR was replicated three times by using the cDNAs from independent experiments. The results of amplification were checked using 1.5% agarose gel electrophoresis. All PCR amplifications were performed using the EasyTaq PCR SuperMix (AS111, TRANS, Beijing, China).

### Quantitative real-time PCR (qPCR)

The transcription levels of *PDS*, *DCL*4, or *AGO*2 genes in the upper leaves inoculated with *CPB1B*-based vectors at 14 dpi were determined using qPCR with primer sets AtPDS-604F/AtPDS-757R, DCL4-363F/DCL4-539R, or AGO2-1575F/AGO2-1754R, respectively (Additional file 1: Table S3). The expression of the *AtActin1* gene determined using the primer set AtActin1-443F/AtActin1-618R (Additional file 1: Table S3) was referred as an internal control to normalize cDNA concentrations. All qPCRs were carried out in three independent biological replicates and triplicate for each cDNA sample by using the SYBR®Premix Ex Taq™ II Kit (Takara) on the StepOne™ Real-Time PCR system (Applied Biosystems), and Data were analyzed using the comparative ΔΔCT method [19]. All values were conducted for at least three biological repeats, and results were presented as mean ± SD. Significant differences between different samples were evaluated with SPSS Statistics 19.0 software.

### Detection of viral RNAs by using RNA blot hybridization

The Northern blot assay was used to detect TCV viral RNAs. Total RNA was extracted as described above. Each total RNA sample (0.8 µg) was separated on 1% agarose gels containing 2% formaldehyde before being transferred to the Hybond-N + membranes (GE Healthcare Life Science). The membranes were incubated with the DIG-labeled DNA probe, which was prepared as follows. A 667 nt fragment of TCV was obtained using PCR with primers TCV-3334F and TCV-4000R (Additional file 1: Table S3). The fragment was used to generate a DIG-dUTP-labeled probe by using the DIG High Prime DNA Labeling and Detection Starter Kit II (Roche). The images of the Nouthern blot results were taken using an ImageQuant LAS4000mini (GE Healthcare Life Science). The total RNA on the agarose gel was stained with ethidium bromide as a loading control.

## Results

### *CPB-CC*-based vector inserting the 46 nt *PDS* fragment in the antisense orientation is more efficient in VIGS than that in the sense orientation

The *PDS* gene encoding phytoene desaturase, a key enzyme in carotenoid biosynthesis, is widely used as a marker for the effectiveness of VIGS because the silencing of *PDS* produces a typical white color due to photobleaching [9, 10, 15, 20]. Previous study showed that the TCV-derived vector *CPB-CC-PDS*, which harboring a 90 nt *PDS* fragment, can induce modest *PDS* silencing in infected plants except *dcl2/4* double mutant [15, 16]. PNRSV-based vectors are reported to harbor foreign inserts that can trigger silencing in the sense orientation [11], whereas the BMV VIGS vector inserting the antisense strand of a gene results in a high degree of silencing [21]. In other studies, the sense or the antisense strand of a gene results in a similar level of BSMV-based VIGS in barley and wheat [22, 23]. These findings prompted us to determine the optimal orientation of the foreign insert in the *CPB-CC-*based VIGS vector. To this end, the 90 nt *PDS* fragment insertted in *CPB-CC-PDS* was subjected to a software to predict the potential siRNAs. One set of short complementary primers, PDS842-887F/PDS883-841R consisting of the predicted siRNA sequence were synthesized (Additional file 1: Table S2). Additional nucleotides “TAC” were added to the 3′ of primer PDS883-841R to facilitate the short complementary primers, thereby producing stacked ends capable of ligating into KpnI-digested *CPB-CC* after the annealing treatment. After ligation, the 5′ proximal (relative to *PDS* fragment) KpnI site was restored, but the 3′ proximal KpnI site was lost. This feature helped the characterization of the orientation of insert and allowed fusing another fragment of the interested target gene in tandem with the *PDS* fragment mentioned above. The resulting *CPB1F* construct inserted in the sense orientation had unique KpnI site at the 5′ of the inserted *PDS* fragment. By contrast, the resulting *CPB1B* construct inserted in the antisense orientation had unique KpnI site at the 3′ of the inserted *PDS* fragment.

Since the albinism induced by *CPB-CC-PDS* in *dcl4* mutant was more obvious than that in wild type [16], *dcl4* mutant plants were used to determine the optimal orientation of the foreign insert in the *CPB-CC*-based VIGS vector. The in vitro transcripts of the resulting constructs, namely, *CPB1F* and *CPB1B*, together with *CPB-CC* were used to infect *dcl4* mutant plants kept at 18 ℃. At 11 dpi, the upper uninoculated leaves of all *CPB1F*- or *CPB1B*-infected plants exhibited the photobleaching phenotype, resulting from the reduction in the expression level of *PDS* (Fig. 1a). The semiquantitative RT-PCR validated that the *PDS* mRNA levels in *CPB1B*- and *CPB1F*-infected plants substantially decreased compared with those in *CPB-CC*-infected plants (Fig. 1c). These results clearly illustrated that the *CPB-CC*-based vectors inserting the 46 nt *PDS* fragment in both orientations could effectively trigger *PDS* silencing in *Arabidopsis*.

### The optimal insertion size of the *CPB1B* VIGS vector is around 100 nt

**Fig. 2:**
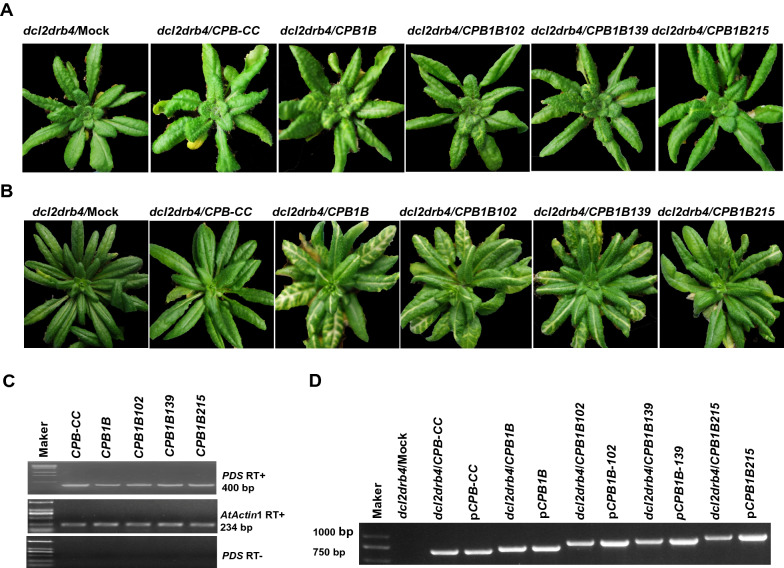
*PDS* silencing in *dcl2drb4* plants inoculated with different *CPB1B*-based VIGS vectors harboring foreign inserts with varying sizes in the antisense orientation. Images of plants recorded at **a** 21 and **b** 38 dpi. At 11 dpi, the upper leaves of *CPB1B*-inoculated plants gradually exhibit photobleaching. Photobleaching is observed with 2 days delay in *CPB1B*102- and *CPB1B*139-infected plants, but this observation is not evident at 18–20 dpi in the *CPB1B*215-infected plants. The photobleaching of all plants inoculated with *CPB1B*215 is observed in the main vein, and only a few lateral veins have exhibited photobleaching. The VIGS persists throughout the plant growth period in infected plants and increases with time, as indicated by the photobleaching. **c** Downregulation of *PDS* mRNA levels by using different *CPB1B*-based VIGS vectors with foreign inserts of varied sizes, as determined using semiquantitative RT-PCR. The samples are collected at 14 dpi. The *AtActin*1 mRNA is used as a control to ensure that similar amounts of RNA are used in all reactions. **d** Conventional RT-PCR of the genetic stability of the foreign insert in the recombinant virus by using primers TCV-3334F/TCV-4000R. The predicted sizes of RT-PCR amplification products derived from plants infected with *CPB-CC*, *CPB1B*, *CPB1B*102, *CPB1B*139, and *CPB1B*215 are 667, 713, 812, 849, and 925 nt, respectively. The RT-PCR products are amplified from all infected samples

Given that different viruses can tolerate foreign inserts in a particular range of sizes, a series of *CPB1B*-derived vectors harboring another *PDS* fragment of varied size in the antisense orientation were further constructed by inserting 102, 139, and 215 nt *PDS* fragment to KpnI-treated *CPB1B*, resulting in CPB1B-derived vectors with one more *PDS* fragment in size of 102, 139, and 215 nt, designated as *CPB1B*102, *CPB1B*139, and *CPB1B*215, respectively. The in vitro transcripts of the resulting constructs were used to infect *dcl2drb4* mutant plants kept at 18 ℃. At 11 dpi, the upper leaves of all plants inoculated with *CPB1B* gradually exhibited photobleaching. The photobleaching was observed with 2 days delay in *CPB1B*102 and *CPB1B*139-infected plants, but this observation was not evident until at 18–20 dpi in the *CPB1B*215-infected plants. The photobleaching of all plants inoculated with *CPB1B*215 was observed in the main vein, and only a few lateral veins exhibited albinism (Fig. 2a, b). The VIGS persisted throughout the plant growth period in the infected plants and increased with time, as indicated by the photobleaching. As shown in Fig. 2a, b, the virus-infected plants generated varying degrees of *PDS* silencing depending on the size of inserts. The virus harboring a long foreign insert induced weak *PDS* silencing. Consistently, as detected by semiquantitative RT-PCR, the mRNA expression levels in *CPB1B*, *CPB1B*102, and *CPB1B*139-infected plants were substantially lower than that in plants infected with *CPB-CC* virus that did not contain the *PDS* insert, an a mild decrease in the mRNA expression level in plants infected with *CPB1B*215 was observed compared with that in plants infected with *CPB-CC* (Fig. 2c). This result confirmed that the photobleaching phenotype was correlated with the silencing of the endogenous *PDS* gene, which served as a visualizable marker to indicate the penetrance of VIGS.

The genetic stability of foreign inserts in these recombinant viruses was evaluated through conventional RT-PCR by using the primers TCV-3334F/TCV-4000R flanking the foreign insert in CPB genomic RNA. The predicted sizes of RT-PCR amplification products derived from plants infected with *CPB-CC*, *CPB1B*, *CPB1B*102, *CPB1B*139, and *CPB1B*215 were 667, 713, 812, 849, and 925 nt, respectively. The predicted RT-PCR products were amplified from all infected samples respectively (Fig. 2d). These data suggested that the *CPB1B*-based VIGS vector could tolerate foreign inserts with size up to 215 nt, whereas harboring 139 or 215 nt foreign inserts substantially affected the movement and the silencing efficiency of the virus, apparently indicating delayed appearance and reduced photobleaching (Fig. 2a, b). The *CPB1B*102 fused with 102 foreign insert did not affect the silencing efficiency substantially, but the movement of the *CPB1B*102 virus (2 days delay) was somewhat slower than that of the original *CPB1B* vector, as indicated by photobleaching. Thus, the optimal insertion size of the VIGS vector was around 100 nt.

### *CPB1B* permits simultaneous silencing of two different *Arabidopsis* genes

**Fig. 3:**
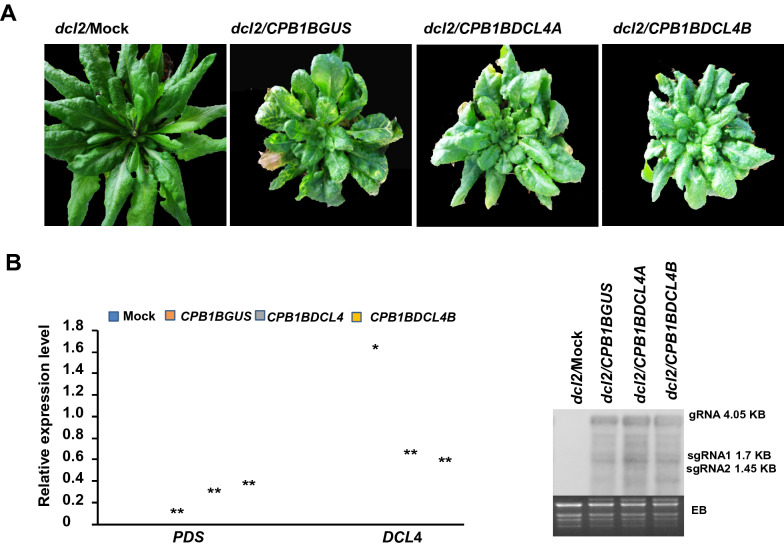
Silencing *PDS* and *DCL*4 gene in *Arabidopsis* simultaneously through the *CPB1B*-based VIGS vectors and its effect on virus replication. **a** Images of plants recorded at 51 dpi. At 51 dpi, the photobleaching is observed in the upper leaves of all plants inoculated with *CPB1BGUS*, and the rosette of *CPB1BGUS*-infected plants is substantially smaller than that of uninfected plants. However, the photobleaching is not observed in the *CPB1BDCL4A*- and *CPB1BDCL4B*-infected plants, but all infected plants show severe symptoms of virus infection as *CPB1BGUS*-infected plants. **b** qRT-PCR analysis of *PDS* and *DCL*4 expression levels in the upper uninoculated leaves of *CPB1B*-based VIGS vector-infected plants at 14 dpi. Expression is normalized against *AtActin1* gene was used as an internal control. All values represent means ± SD from three independent biological replicates and asterisks denote significantly different from the control group (*P < 0.05, **P < 0.01). **c** The viral RNA accumulation levels in the upper uninoculated leaves of *CPB1B*-based VIGS vector-infected plants at 14 dpi. *EB* ethidium bromide-stained Northern gel, *gRNA* genomic RNA, *sgRNA* subgenomic RNA

The efficiency of *CPB1B*-VIGS as a novel tool in the reverse genetics studies in *Arabidopsis* was further evaluated by silencing the *DCL4* gene, a primary DCL in *Arabidopsis*. Two pieces of *DCL*4 fragments with size of 100 nt consisting of at least one of the top five predicted siRNA were selected for cloning into the KpnI-treated *CPB1B*. Similarly, *CPB1BGUS* with the same size as the GUS gene fragment was generated and served as the no-target control. The in vitro transcripts of the resulting constructs were then used to infect *dcl2* mutant plants kept at 18 ℃. At 13 dpi, the photobleaching, which resulted from the downregulation of the *PDS* gene, was observed in the upper leaves of all plants inoculated with *CPB1BGUS*, and the rosette of the *CPB1BGUS*-infected plants was substantially smaller than that of the uninfected plants (Fig. 3a). These results indicated that *CPB1BGUS* could silence the *PDS* gene effectively. However, the photobleaching was not obvious in the *CPB1BDCL4A*- and the *CPB1BDCL4B* infected plants even though the symptom of virus infection were as severe as those *CPB1BGUS*-infected plants (Fig. 3a).

The mRNA expression levels of the *PDS* and the *DCL4* genes were detected using qRT-PCR at 14 dpi. As shown in Fig. 3b, the *PDS* transcript levels were downregulated in *CPB1BGUS*-, *CPB1BDCL4A*-, and *CPB1BDCL4B*-infected plants relative to that of uninfected healthy plants. Consistent with the photobleaching phenotype, the extent of reduction in *CPB1BDCL4A*- or *CPB1BDCL4B*-infected plants was less than that in *CPB1BGUS*-infected plants. qRT-PCR also revealed that the relative amount of *DCL*4 transcripts in *CPB1BDCL4A*- and *CPB1BDCL4B*-infected plants were substantially lower than those in uninfected plants, whereas the abundance of *DCL*4 mRNA in *CPB1BGUS*-infected plants increased significantly. These results indicated that *CPB1B*-derived vectors could silence *PDS* and the target gene inserted in tandem simultaneously. Inoculation with *CPB1BGUS*, the no-target control, could stimulate the expression of *DCL*4, implicating that DCL4 was involved in the antivirus defense against TCV. This finding was consistent with those reported in previous studies [14, 17, 24].

The effect of silencing the *DCL*4 gene on virus replication was further evaluated by monitoring the TCV viral RNA. The upper uninoculated leaves were collected from the *CPB1BDCL4A*-, *CPB1BDCL4B*-, *CPB1BGUS*-infected plants and uninfected plants at 21 dpi and subjected to RNA extraction and Northern blot hybridization with TCV-specific probes. As shown in Fig. 3c, compared with *CPB1BGUS*-infected plants, the *CPB1BDCL4A*- and the *CPB1BDCL4B*-infected plants had substantially increased TCV viral RNA levels, in which the *DCL*4 gene was downregulated substantially. This result revealed that *DCL*4 knockdown could elevate the replication of TCV, which indicated that DCL4 was involved in the antivirus defense against TCV. This result agreed with those of previous studies, which showed that DCL4 has a critical role in antiviral defense [18, 24]. Thus, *CPB1B* VIGS can be used as a novel tool for the functional characterization of the target gene in *Arabidopsis* with visualizable indicator of the penetrance of VIGS.

### AGO2 involved in the DRB4-independent DCL4-mediated *PDS* silencing

**Fig. 4:**
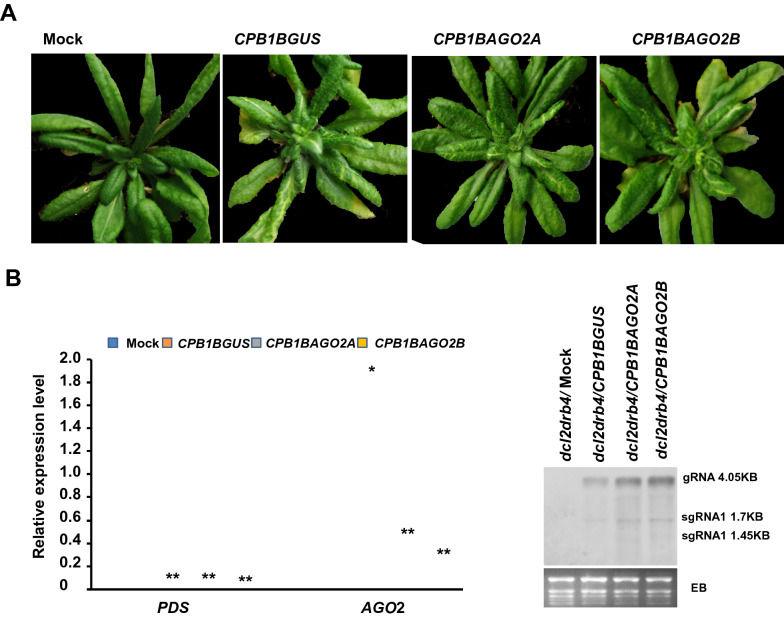
*AGO2* gene involved in the DRB4-independent DCL4-mediated antiviral defense in *Arabidopsis*. **a** Images of plants recorded at 25 dpi. At 13 dpi, the photobleaching is observed in the upper leaves of all plants inoculated with *CPB1B*-based VIGS vectors. **b** qRT-PCR analysis of *PDS* and *AGO*2 expression levels in the upper uninoculated leaves of *CPB1B*-based VIGS vector-infected plants at 14 dpi. Expression is normalized against *AtActin1* gene was used as an internal control. All values represent means ± SD from three independent biological replicates and asterisks denote significantly different from the control group (*P < 0.05, **P < 0.01). **c** The viral RNA accumulation levels in the upper uninoculated leaves of *CPB1B*-based VIGS vector-infected plants at 14 dpi. *EB* ethidium bromide-stained Northern gel, *gRNA*: genomic RNA, *sgRNA* subgenomic RNA

Previous study has shown a substantial subset of the DCL4 antiviral activity, which is DRB4-independent indicating by *dcl2drb4* double knockouts caused a far smaller loss of antiviral silencing than *dcl*2*dcl*4 double knockouts and *CPB-CC-PDS* can induce *PDS* silencing in *dcl2drb4* plants but not in *dcl2dcl*4 double knockout [16]. AGOs are the effector proteins in eukaryotic small RNA (sRNA)-based gene silencing pathways controlling gene expression, transposon activity, and antivirus defense [25]. To investigate whether AGO2 gene involved in this DRB4-independent DCL4-mediated antiviral defense in *dcl2drb4* mutant, *CPB1B*-based VIGS vectors which with one piece of the 100 nt *AGO*2 gene fragment, *CPB1BAGO2A* and *CPB1BAGO2B*, were generated using the method mentioned above. The in vitro transcripts of these two *CPB1B*-derived vectors and *CPB1BGUS* as well which serving as control were used to infect *dcl2drb4* double knockout mutant plants kept at 18 ℃. Photobleaching was observed at 13 dpi, which indicated that the penetrance of VIGS in virus-inoculated plants (Fig. 4a). qRT-PCR was used to verify the silencing of the *PDS* gene. As shown in Fig. 4b, the mRNA expression level of *PDS* in virus-inoculated plants decreased substantially compared with that in uninfected plants. qRT-PCR also revealed that the abundance of *AGO2* mRNA in *CPB1BAGO2A*- and *CPB1BAGO2B*-infected plants decreased profoundly compared with that in uninoculated or *CPB1BGUS*-infected plants (Fig. 4b). These results reconfirmed that the *CPB1B*-derived vector could silence *PDS* and the targeted gene inserted in tandem simultaneously and that photobleaching could serve as a gene silencing indicator. However, the expression level of the *AGO2* gene increased in plants inoculated with *CPB1BGUS* with 100 nt *GUS* gene, which was not an *Arabidopsis* endogenous gene. This result implicated that the *AGO*2 was involved in the antiviral defense in the *dcl2drb4* double mutant.

The upper uninoculated leaves were collected from the *CPB1BAGO2A*-, *CPB1BAGO2B*-, and *CPB1BGUS*-infected plants and uninfected plants at 21 dpi and subjected to RNA extraction and Northern blot hybridization with TCV-specific probes to corroborate the function of AGO2 in the antivirus defense in *dcl2drb4* double mutant. As shown in Fig. 4c, compared with those in *CPB1BGUS*-infected plants, the TCV viral RNA levels in *CPB1BAGO2A*- and *CPB1BAGO2B*-infected plants increased substantially. These results revealed that the *AGO2* knockdown could facilitate the replication of TCV, indicating that the involvement of AGO2 in *PDS* silencing in the *dcl2drb4* mutant. Collectively, these results demonstrated the *CPB1B*-based VIGS system as a valuable tool with visualizable indicator of VIGS for interrogating *Arabidopsis* genes, especially those involved in the RNA silencing pathways.

## Discussion and conclusions

VIGS is an attractive reverse genetics tool for functional genomics in plants. Therefore, in the past decade, tremendous improvements in VIGS have been reported [4–8, 12]. Efforts has been made to select the efficient reporter gene [10, 26–28]. As far as we know, VIGS vectors with traceable and visualizable indicator for the prediction of positive gene silencing plants for reverse genetics are still lacking. In this study, we have developed a novel VIGS vector, designated *CPB1B*, based on an attenuated variant of *Turnip crinkle virus* (TCV) known as CPB. *CPB1B* induced robust PDS silencing, causing easily visible photobleaching in systemically infected *Arabidopsis* leaves, which provides a preliminary assessment and traceable marker of the silencing penetrance. More importantly, *CPB1B* can accommodate additional insert leading the silencing of two different Arabidopsis genes simultaneously. The VIGS persists throughout the plant growth period in the infected plants and increases with time, as indicated by the photobleaching.

TCV is a small icosahedral plant virus with a (+)-strand RNA genome encoding five proteins. *CPB-CC-PDS*, a TCV derivative vector bearing 90 nt *PDS* fragment, can induce modest *PDS* silencing, providing a visual indicator for the gene silencing [15, 31]. Studies show that the antiviral RNA silencing in plants enlists DCL2 and DCL4, DCL3 to a lesser extent, to process the dsRNA of virus origin into viral siRNAs (vsiRNAs) with size of 21–24 nt [2, 15, 18, 24]. Theoretically, the insertion of a 21–24 nt fragment can induce silencing. To obtain a short PDS fragment as a reporter gene, we have predicted the potential siRNA sequence in the 90 nt *PDS* insert in CPC-CC-PDS [15] and generated two constructs with the same 46 nt *PDS* fragment while inserted in different orientation. The inoculated plants with in vitro transcripts of the resulting constructs can effectively trigger *PDS* silencing in *Arabidopsis* and the silencing efficiency of *CPB1B* with foreign fragment inserted in the antisense orientation is more effective than that of CPB1F with foreign fragment inserted in the sense orientation. However, constructs possess the left sequences of the 90 nt *PDS* (Additional file 1: Table S1), designated CPB2F and CPB2B, which does not contain any predicted siRNA sequence cannot induce *PDS* silencing efficiently (Additional file 1: Fig. S2). Thus, the selection of the inserting fragment with predicted siRNA sequence is critical in the development of a VIGS vector with high gene silencing efficiency.

RNA viruses have optimal genome capacity for efficient replication and virion assembly. Therefore, virus-based vectors have restrictions in carrying and expressing heterologous sequences in accordance with their genome capacity [29–31]. As shown in Fig. 2, although the inserted fragments of *CPB1B*102 and *CPB1B* have the same predicted siRNA sequences (Additional file 1: Fig. S2), the silencing efficiency induced by *CPB1B*102 is lower than that of *CPB1B*. Furthermore, *CPB1B*139 triggered less *PDS* silencing than *CPB1B*102 did, even though its inserting has one more predicted siRNA sequence than that of *CPB1B*102 (Additional file 1: Fig. S2). We have speculated that the increased insert length affects the movement of the virus, thereby affecting the efficiency of silencing. Thus, to develop efficient VIGS vector minimizing the size of inserting fragment is important too. In the present work, we have demonstrated that *CPB1B* and *CPB1F* with 46 nt *PDS* fragment could trigger *PDS* silencing efficiently. These results open a potential avenue for the development of VIGS vector through the synthesis of short fragment, which includes several predicted siRNA sequences at the same time. Further attempts should be made to develop efficient VIGS vector through synthetic fragment which could silence two or more functional genes simultaneously.

The RNase III enzyme Dicer is essential for the initiation of RNA silencing [1, 2, 15, 18, 24]. The efficiency of *CPB1B*-based VIGS as a novel efficient tool for reverse genetics studies in *Arabidopsis* is evaluated by silencing the *DCL4* gene in the *dcl*2 knockout mutant. Results show that *CPB1B*-based VIGS vectors with *DCL*4 insert, namely, *CPB1BDCL4A* and *CPB1BDCL4B*, can knock down *DCL*4 efficiently (Fig. 3b), resulting in the increased accumulation of viral RNA (Fig. 3c). Since *DCL2* has been knocked out in *dcl2* mutant, so the increased accumulation of viral RNA is due to the knock down *DCL*4. These findings are consistent with those reported in a previous study that DCL4 is a key factor of *Arabidopsis* in antiviral defense. However, no apparent photobleaching is observed in *DCL*4-downregulated *dcl*2 plants despite the high levels of viral RNA, which is similar to the phenotype of *dcl2dcl4* double knockout mutant infected with *CPB-CC-PDS* [15, 16]. The antiviral RNA silencing in plants enlists DCL2 and DCL4, DCL3 to a lesser extent, to process the dsRNA of virus origin into vsiRNAs [15, 16, 18, 24]. Thus, the infected *dcl*2 plants cannot produce enough vsiRNA to silence *PDS* when *DCL*4 is down regulated despite the high levels of viral RNA. Collectively, these results validate the involvement of *DCL4* in *PDS* silencing.

AGOs are the effector proteins in eukaryotic siRNA-based gene silencing pathways controlling gene expression, transposon activity, and antivirus defense [25]. Among the 10 AGO genes encoded by the *Arabidopsis* genome, AGO1, AGO2, and AGO7 are demonstrated to be involved in antiviral defense against TCV [15, 18]. The application of the *CPB1B*-based VIGS system developed in the present work shows that the AGO2 gene is involved in *PDS* silencing in *dcl2drb4* double mutant. As illustrated in Fig. 3, qRT-PCR reveals that the abundance of the *AGO2* mRNA decreases profoundly in *dcl2drb4* plants infected with the VIGS vector with the *AGO2* gene insert, namely, *CPB1BAGO2A* or *CPB1BAGO2B*, compared with that in uninoculated plants or plants infected with *CPB1BGUS*, which is a nontarget VIGS control. Northern blot shows that the downregulation of the *AGO2* gene increases the accumulation of the TCV viral RNA, indicating that AGO2 is involved in the DCL4-mediated antiviral defense in *dcl2drb4* plants. Since DRB4 which commonly partners with DCL4 in the antiviral silencing pathway has been knocked out in *dcl2drb4* plants, this results also confirmed that DRB4 was dispensable for *PDS* silencing induced by *CPB1B* derivatives.

In summary, the *CPB1B*-based vector developed in this work is an efficient novel tool with visualizable indicator that permits a preliminary assessment of the silencing penetrance, for interrogating *Arabidopsis* genes especially those involved in the RNA silencing pathways. In addition, the development of the VIGS vector by inserting a synthetic fragment consisting of predicted siRNA sequence opens a potential avenue for the development of VIGS vector through the synthesis of short fragments, which include several predicted siRNA sequences to silence two or more functional genes simultaneously.

## Supplementary Information

**Additional file 1: Figure S1.** Phenotype of *dcl4* inoculated with *CPB2F* and *CPB2B*. **Figure S2.** Sequence of *PDS* insert in the *CPB1B* VIGS vector. **Table S1.** Fragments selected to develop VIGS vectors. **Table S2.** Primers used for plasmid construction. **Table S3.** Primers used for RT-PCR, semiquantitative RT-PCR, and quantitative RT-PCR.

## Data Availability

The material used during the current study are available from the corresponding author on reasonable request.
