# Supplementary material for: Simultaneous silencing of two different Arabidopsis genes with a novel virus-induced gene silencing vector
Source: Plant Methods. 2021 Jan 6;17:6. doi: 10.1186/s13007-020-00701-6 (PMC7788715; doi:10.1186/s13007-020-00701-6)
Supplement: Supplementary file 1 — Additional file 1: Figure S1. Phenotype of dcl4 inoculated with CPB2F and CPB2B. Figure S2. Sequence of PDS insert in the CPB1B VIGS vector. Table S1. Fragments selected to develop VIGS vectors. Table S2. Primers used for plasmid construction. Table S3. Primers used for RT-PCR, semiquantitative RT-PCR, and quantitative RT-PCR. [file 13007_2020_701_MOESM1_ESM.docx]

**
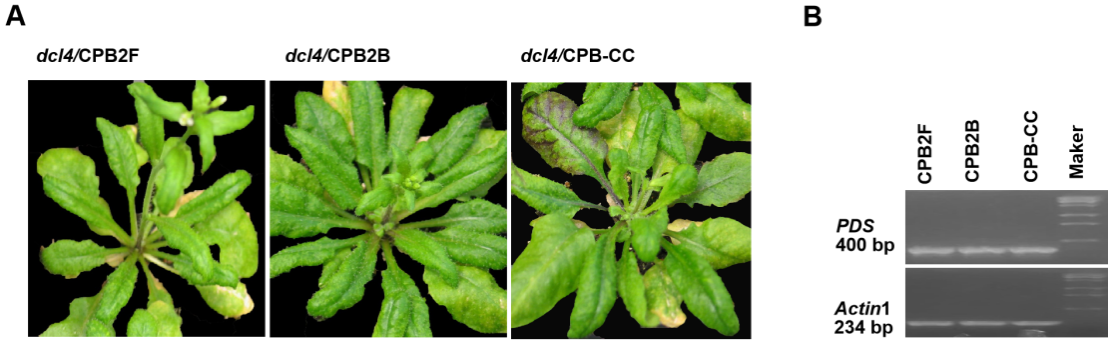
**

**Figure S1 Phenotype of *dcl4* inoculated with *CPB2F* and *CPB2B***

1. *CPB-CC*-based vectors harboring 42 nt *PDS* fragment without predicted siRNA sequence cannot trigger *PDS* silencing in *Arabidopsis*. Images are recorded at 25 dpi. (B) Downregulation of *PDS* mRNA levels by using different CPB1B-based VIGS vectors with foreign inserts of varied sizes, as determined using semiquantitative RT-PCR. The samples are collected at 14 dpi. The *AtActin*1 mRNA is used as a control to ensure that similar amounts of RNA are used in all reactions.


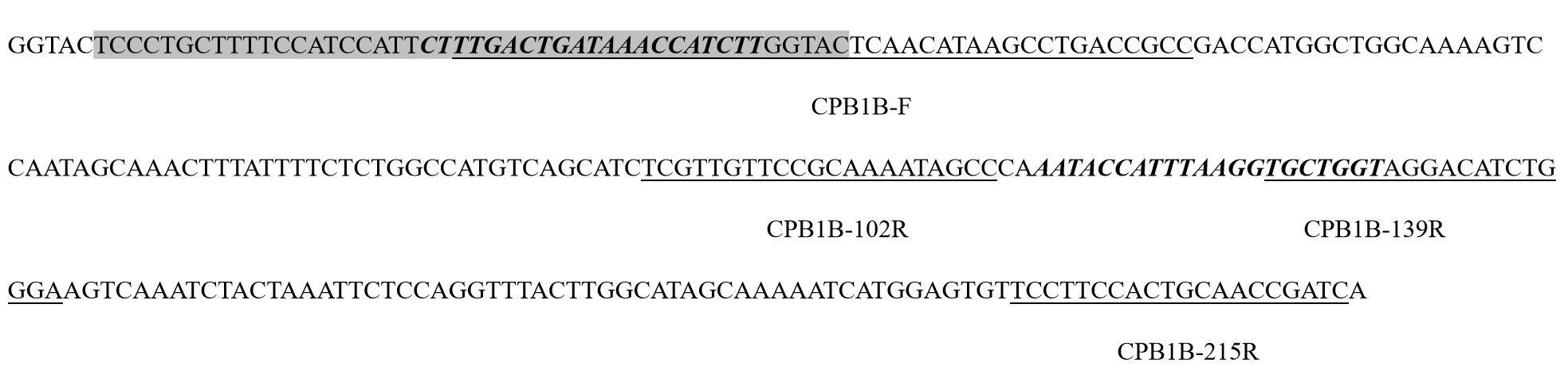


**Figure** S**2** **Sequence of *PDS* insert in the *CPB1B* VIGS vector**

Shaded letters indicate the sequence of primer PDS883-841R, and underlined sequences are primers used to amplify the *PDS* fragment with different sizes. Bold italic shaded letters indicate the predicted siRNA sequences analyzed using the <https://www.genscript.com/tools/sirna-target-finder> software.

Table S1: Fragments selected to develop VIGS vectors

| Fragment | Sequence（5′–3′） | VIGS vector |
| --- | --- | --- |
| PDS1 | CCC***AAGATGGTTTATCAGTCAAAG***AATGGATGGAAAAGCAGGGAGTAC | *CPB1F* and *CPB1B* |
| PDS2 | CTGAGCGCGTGACCGACGAGGTGTTTATTGCCATGTCAAAGG | *CPB2F* and CPB2B |
| GUS | GT***CACGCGCTATCAGCTCTTTAA***TCGCCTGT***AAGTGCGCTTGCTGAGTTTCC***CCGTTGACTGCCTCTTCGCTGTACAGTTCTTTCGGCTTGTTGCCCGCT | *CPB1BGUS* |
| DCL4A | ATCAACATTTACA***CCAATCCACTTCAGAAATT***TCACAGCACCTTTGAAGCCACTGTCAACTAAGAAAGCTCCCAC***AAGAGCCTCAACCACATCAGC***AAT | *CPB1BDCL4A* |
| DCL4B | C***AACTCTCCTTCATCAAGACTA***TCATGGTGTAGAAAAAGGTGTCGGCTAACAGCAAACTTGAGGAATGCATCACC***AAGCACCTCAAGCCTTTCAAG***AGAAAGGCGCTC | *CPB1BDCL4B* |
| AGO2A | TCTC***AAGCTCTGCATGCAGCTTGAA***AATCGCTTTGTCCTCAATTGTAAGAGAAGATAATGAAGAGGC***AAGCGAAGCAGCAGAGGTTGA***CGCTCCCCGCGG | *CPB1BAGO2A* |
| AGO2B | GGCGGAACAAGAGAGACGGGTTTGGTGCAGCGAGTGA***AAGTGAAGCACATCTCGAAGA***TGAGCTTCTGCACCTGATCCGAAGTGAATCCAAGTTCGTCCC | *CPB1BAGO2B* |

^a^ Bold italic letters indicate the predicted siRNA sequences analyzed using the <https://www.genscript.com/tools/sirna-target-finder> software.

Table S2: Primers used for plasmid construction

| Primer | Sequence（5′–3′） |
| --- | --- |
| PDS842-887F | C***AAGATGGTTTATCAGTCAAAG***AATGGATGGAAAAGCAGGGAGTAC |
| PDS883-841R | TCCCTGCTTTTCCATCCATTCTTTGACTGATAAACCATCTTGGTAC |
| PDS888-928F | CTGAGCGCGTGACCGACGAGGTGTTTATTGCCATGTCAAAGTAC |
| PDS927-884R | TTTGACATGGCAATAAACACCTCGTCGGTCACGCGCTCAGGTAC |
| CPB1B-F | ttgactgataaaccatcttggtacTCAACATAAGCCTGACCGCC |
| CPB1B-102R | ctaagatgagaagactacactatgGGCTATTTTGCGGAACAACGA |
| CPB1B-139R | ctaagatgagaagactacactatgTCCCAGATGTCCTACCAGCA |
| CPB1B-215R | ctaagatgagaagactacactatgGATCGGTTGCAGTGGAAGGA |
| Gus-F | ttgactgataaaccatcttggtacGTCACGCGCTATCAGCTCTTTAATC |
| Gus-R | ctaagatgagaagactacactatgAGCGGGCAACAAGCCGAAAGAACT |
| AtDcl4A-F | ttgactgataaaccatcttggtacATCAACATTTACACCAATCCACTTC |
| AtDcl4A-R | ctaagatgagaagactacactatgATTGCTGATGTGGTTGAGGCTCTTG |
| AtDcl4B-F | ttgactgataaaccatcttggtacCAACTCTCCTTCATCAAGACTATCATG |
| AtDcl4B-R | ctaagatgagaagactacactatgCGCCTTTCTCTTGAAAGGCTTG |
| AGO2A-F | ttgactgataaaccatcttggtacTCTCAAGCTCTGCATGCAGCTTG |
| AGO2A-R | ctaagatgagaagactacactatgCCGCGGGGAGCGTCAACCTCT |
| AGO2B-F | ttgactgataaaccatcttggtacGGCGGAACAAGAGAGACGGGT |
| AGO2B-R | ctaagatgagaagactacactatgGGGACGAACTTGGATTCACTTC |
| AGO2B-R | ctaagatgagaagactacactatgGGGACGAACTTGGATTCACTTC |

^a^ Bold italic letters indicate the predicted siRNA sequences analyzed using the <https://www.genscript.com/tools/sirna-target-finder> software.

^b^ Lowercase letters indicate the overlap sequence of *CPB1B* treated with *Kpn* I used for seamless assembly.

Table S3: Primers used for RT-PCR, semiquantitative RT-PCR, and quantitative RT-PCR

| Primer | Sequence（5′–3′） |
| --- | --- |
| TCV-3334F | GCGGATGGTATCAGCGATCCA |
| TCV-4000R | GTCACCACAGCCCACCCTTTC |
| AtActin1-F | GGGCAAGTGATCACCATTGG |
| AtActin1-R | TGGAGCCAAAGCAGTGATCTC |
| AtPDS-1200F | CCTTCCATGGTGTTTGCCGCTCCAGTCGATA |
| AtPDS-1600R | GGAATTTGGCCATCTTGGAGTCTTAACGACATGGTA |
| AtActin1-443F | GTCGTACTACCGGTATTGTGC |
| AtActin1-618R | TGCTGTGGTGGTGAAAGAGT |
| AtPDS-604F | GGTATTTGGGCTATTTTGCG |
| AtPDS-757R | CTCCCTGCTTTTCCATCCA |
| DCL4-363F | AAAAATCGCCAGGAGGTATC |
| DCL4-539R | ACGGTGGGAGCAAGAAAAA |
| AGO2-1575F | CGGTGGTGAAATCATTGGAA |
| AGO2-1754R | CCCCTTGTGACTCCCTTCTT |
